# Characterization of a novel low-temperature-active, alkaline and sucrose-tolerant invertase

**DOI:** 10.1038/srep32081

**Published:** 2016-08-24

**Authors:** Junpei Zhou, Limei He, Yajie Gao, Nanyu Han, Rui Zhang, Qian Wu, Junjun Li, Xianghua Tang, Bo Xu, Junmei Ding, Zunxi Huang

**Affiliations:** 1 Engineering Research Center of Sustainable Development and Utilization of Biomass Energy, Ministry of Education, Yunnan Normal University, Kunming, 650500, People’s Republic of China; 2 College of Life Sciences, Yunnan Normal University, Kunming, 650500, People’s Republic of China; 3 Key Laboratory of Yunnan for Biomass Energy and Biotechnology of Environment, Yunnan, Kunming, 650500, People’s Republic of China; 4 Key Laboratory of Enzyme Engineering, Yunnan Normal University, Kunming, 650500, People’s Republic of China

## Abstract

A glycoside hydrolase family 32 invertase from *Bacillus* sp. HJ14 was expressed in *Escherichia coli*. The purified recombinant enzyme (rInvHJ14) showed typical biochemical properties of low-temperature-active and alkaline enzymes: (i) rInvHJ14 was active and stable in the range of pH 7.0–9.5 with an apparent pH optimum of 8.0; (ii) rInvHJ14 was most active but not stable at 30–32.5 °C, with 19.7, 48.2 and 82.1% of its maximum activity when assayed at 0, 10 and 20 °C, respectively, and the *E*_a_, ΔG^*^ (30 °C), *K*_m_ (30 °C) and *k*_cat_ (30 °C) values for hydrolysis of sucrose by rInvHJ14 was 47.6 kJ mol^−1^, 57.6 kJ mol^−1^, 62.9 mM and 746.2 s^−1^, respectively. The enzyme also showed strong sucrose tolerance. rInvHJ14 preserved approximately 50% of its highest activity in the presence of 2045.0 mM sucrose. Furthermore, potential factors for low-temperature-active and alkaline adaptations of rInvHJ14 were presumed. Compared with more thermostable homologs, rInvHJ14 has a higher frequency of glycine residues and a longer loop but a lower frequency of proline residues (especially in a loop) in the catalytic domain. The catalytic pockets of acid invertases were almost negatively charged while that of alkaline rInvHJ14 was mostly positively charged.

Sucrose can be hydrolyzed by invertases (EC 3.2.1.26) into an equimolar mixture of d-glucose and d-fructose. Invertases, also termed as β-fructofuranosidase and sucrase, fall into glycoside hydrolase (GH) family 32 based on their amino acid sequences homology^1^. Invertases are of great interest in basic research and various industrial applications especially in food industry^2^. These enzymes are used for the preparation of invert sugar, high fructose syrup, jams, candies, soft centered chocolates and cookies, creams, marshmallows, powder milk for infants, artificial honey and beverages items^2^. In addition to sucrose, some invertases also hydrolyze fructose-containing oligosaccharides and fructans, such as raffinose^3^,^4^,^5^,^6^,^7^, stachyose^8^,^9^, kestose^10^,^11^, nystose^10^,^11^, inulin^2^,^11^,^12^,^13^ and levan^14^.

It is well-known that low temperatures strongly inhibit the rates of enzyme-catalysed reactions. Low-temperature-active enzymes are the exceptions because they can show high catalytic activity at low temperatures associated with low stability at intermediate and high temperatures^15^. These properties are of relevance to applications in industries as diverse as food, household detergents, aquaculture, bioremediation, tanning, energy, pharmaceutical and molecular biology^16^. For example, low-temperature-active enzymes can be used in food industry for meat tenderizing, cheese ripening, dough fermentation, wine and beverage stabilization, and clarification of fruit, vegetable juices and wine^16^. Low temperatures avoid spoilage and change in nutritional value and flavour of the original heat-sensitive substrates and products^16^. Biotechnological processes performed at low temperatures can also provide economic benefit by reducing energy consumption^16^. Furthermore, low-temperature-active enzymes are important to maintain the metabolic rates and sustained growth compatible with life in cold ecosystems that actually occupy over 85% of the Earth’s surface^15^. However, low-temperature-active invertase has not been reported to date.

Invertases have been isolated from a variety of sources, including bacteria^17^, yeast^12^, fungi^10^, higher plants^18^ and animals^19^. Acid invertases have been the most extensively studied and isolated from various sources while much less is known about alkaline invertases which have been reported only from plants and cyanobacteria^2^,^4^,^5^,^20^,^21^,^22^,^23^,^24^.

In this study, a new GH 32 invertase, designated InvHJ14, was discovered from *Bacillus* sp. HJ14. The enzyme was expressed in *Escherichia coli* and the recombinant enzyme was purified. The recombinant InvHJ14 (rInvHJ14) was tolerant to sucrose and showed typical properties of low-temperature-active and alkaline enzymes. The potential molecular adaptations to low-temperature and alkaline environments of the enzyme were presumed.

## Results

### Sequence analysis

**Figure 1:**
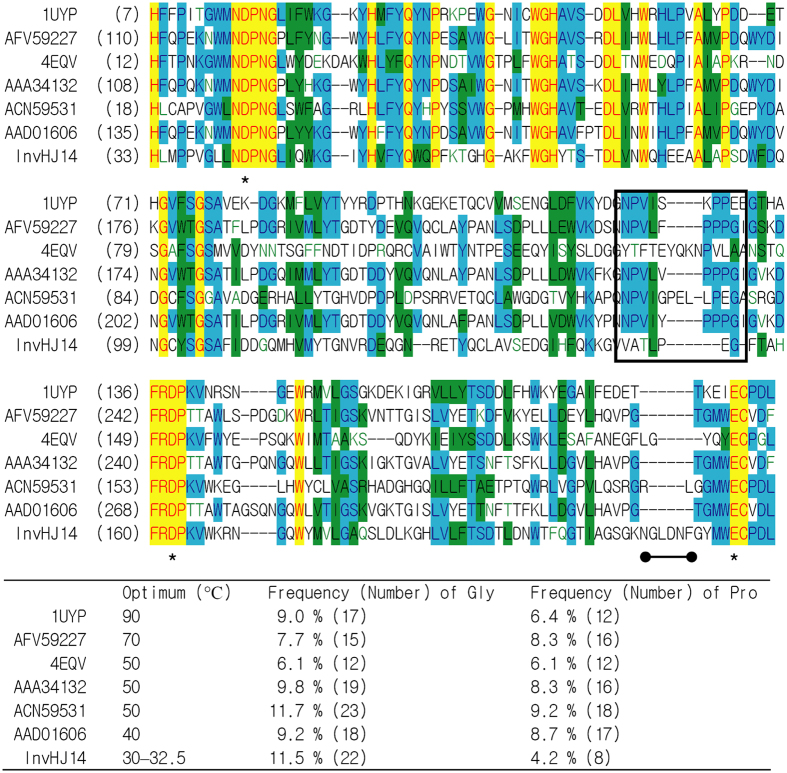
Partial amino acid sequence alignment of InvHJ14 with mesophilic and thermophilic GH 32 invertases. The invertases include InvTM from *T. maritima* (Accession No. or PDB ID: 1UYP)^13^, InvEH from *E. haichowensis* (AFV59227)^38^, InvSC from *S. cerevisiae* (4EQV)^27^, InvSL from *S. lycopersicum* (AAA34132)^39^, InvUB from an uncultured bacterium (ACN59531)^24^ and InvIB from *I. batatas* (AAD01606)^28^. *Asterisks* show the putative active residues. The segment corresponding to V149–G155 of InvHJ14, which presents the difference of proline residues between InvHJ14 and more thermostable homologs, is *framed*. The amino acid residues N210 to F215 of InvHJ14, which build a longer loop in Fig. 2, are *underlined*.

A gene of 1,458 bp encoding putative invertase of 55.9 kDa was predicted from the draft genome of HJ14. *In silico* analysis indicated that the invertase lacked recognizable signal peptide sequence but was comprised of a catalytic domain of GH 32 from H33 to P339 and a C-terminal domain of GH 32 from K338 to Q480. The consensus pattern of GH 32, H-x(2)-[PV]-x(4)-[LIVMA]-N-D-P-N-[GA] (http://prosite.expasy.org/PS00609), was also detected in InvHJ14 (HLMPPVGLLNDPNG in Fig. 1). The residues D43, D162 and E220 are the predicted nucleophile, transition-state stabilizer and general acid/base catalyst, respectively (Fig. 1)^11^,^25^.

A BLASTP search against NCBI protein database showed that InvHJ14 was most similar to a number of putative invertases translated from genome sequences. In the result, InvHJ14 was found to have the maximal sequence identity of 99.6% with the uncharacterized GH 32 invertase from *Bacillus safensis* JPL_MERTA8-2 (WP_046312845). Furthermore, the BLASTP analysis showed that InvHJ14 had 45.6% identity with the experimentally characterized GH 32 invertase (SurA) from *Geobacillus stearothermophilus* NUB36 (AAB38976)^17^.

### Structure analysis

**Figure 2:**
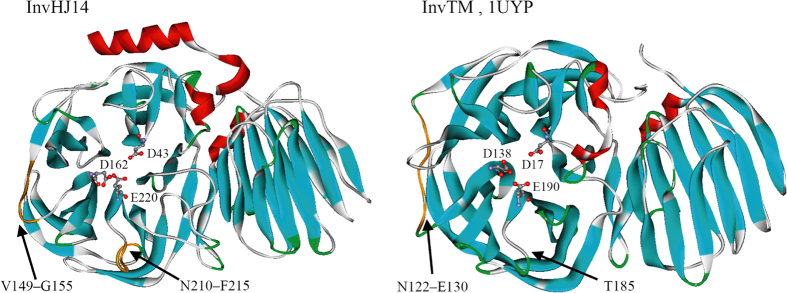
Structures of low-temperature-active InvHJ14 and the thermophilic invertase InvTM. InvTM is a *T. maritima* invertase (Accession No. or PDB ID: 1UYP) which shows optimal activity at 90 °C^13^. The putative active residues are detailed in *ball-and-stick* form. *Arrows* indicate loops built by V149–G155 and N210–F215 of InvHJ14 and the corresponding amino acid residues of InvTM as shown in Fig. 1.

**Figure 3:**
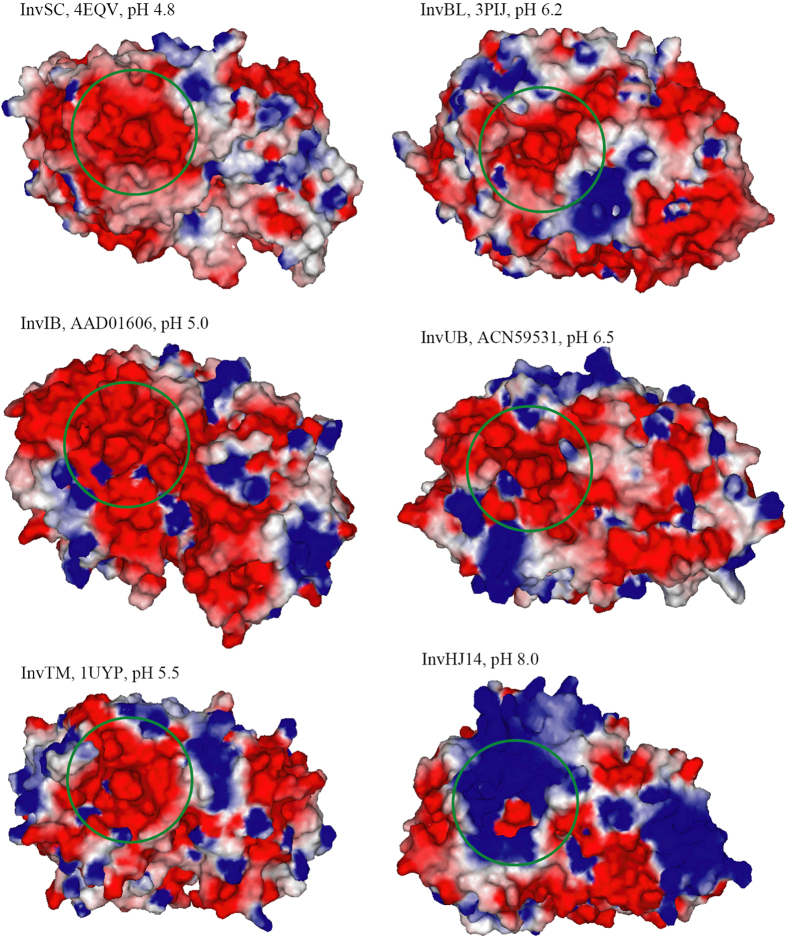
Charge distributions on the surfaces of GH 32 invertases with acidic and alkaline pH optima. The invertases include InvSC from *S. cerevisiae* (Accession No. or PDB ID: 4EQV)^27^, InvIB from *I. batatas* (AAD01606)^28^, InvTM from *T. maritima* (1UYP)^13^, InvBL from *B. longum* (3PIJ)^26^ and InvUB from an uncultured bacterium (ACN59531)^24^. Catalytic pockets are *circled*. Charge distribution on the surface was calculated at pH 7.0. Positive charges are depicted in *blue* and negative charges in *red*.

The acidic invertases InvSC from *Saccharomyces cerevisiae* (Accession No. or PDB ID: 4EQV), InvIB from *Ipomoea batatas* (AAD01606), InvTM from *Thermotoga maritima* (1UYP), InvBL from *Bifidobacterium longum* (3PIJ) and InvUB from an uncultured bacterium (ACN59531) exhibite optimal activities at pH 4.8, pH 5.0, pH 5.5, pH 6.2 and pH 6.5, respectively^13^,^24^,^26^,^27^,^28^. The model of InvIB was built using the crystal structure of a GH 32 β-fructofuranosidase from *Arabidopsis thaliana* (PDB ID: 2QQV) as the template with a sequence similarity of 42% and a GMQE score of 0.63. Models of InvUB and InvHJ14 were built using the crystal structure of InvBL as the template (Fig. 2), with sequence similarities of both 36% and GMQE scores of 0.67 and 0.66, respectively. As shown in Fig. 3, catalytic pockets of these invertases presented different surface charges. The catalytic pockets of acidic InvSC, InvIB, InvTM, InvBL and InvUB were almost negatively charged, while the opposite was observed for InvHJ14.

### Expression, purification and identification of rInvHJ14

rInvHJ14 was successfully expressed in *E. coli* BL21 (DE3), extracted by cell lysis using sonication, and purified by Ni^2+^-NTA metal chelating affinity chromatography. A clear single band (approximately 58 kDa) representing the recombinant invertase was seen on the SDS–PAGE (Fig. S1). MALDI–TOF MS spectrum of the band matched the molecular masses of internal peptides from rInvHJ14 (Fig. S2), confirming that the purified enzyme is indeed rInvHJ14.

### Substrate specificity

Determined at pH 8.0 and 30 °C, the specific activities of purified rInvHJ14 toward 0.5% (w/v) sucrose, raffinose and fructooligosaccharides mixture were 155.1 ± 1.5, 4.5 ± 0.1 and 3.1 ± 0.3 U mg^−1^, respectively. However, no activity of rInvHJ14 was detected toward substrate of 0.5% (w/v) sucralose, stachyose, levan, inulin, starch, birchwood xylan, beechwood xylan, wheat flour arabinoxylan or *p*-nitrophenyl-β-d-xylopyranoside.

### Biochemical characterization

**Figure 4:**
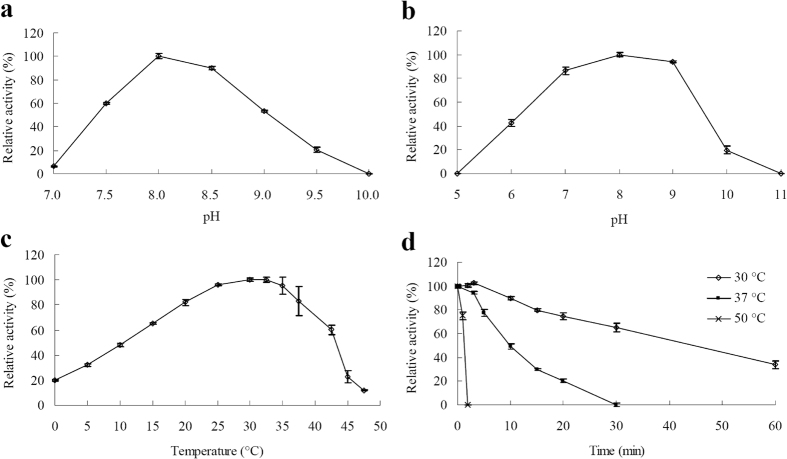
Enzymatic properties of purified rInvHJ14. **(a)** pH-dependent activity profiles. **(b)** pH stability assay. **(c)** Temperature-dependent activity profiles. **(d)** Thermostability assay. *Error bars* represent the means ± SD (n = 3).

Purified rInvHJ14 had activity in the range of pH 7.0–9.5 with an apparent pH optimum of 8.0 (Fig. 4a). Over 85% of its initial activity was observed after incubating the enzyme for 1 h in buffers with pH 7.0–9.0 (Fig. 4b). The apparent optimal temperature for rInvHJ14 was in the range from 30 to 32.5 °C (Fig. 4c). The enzyme could retain 48.2 and 82.1% of its maximum activity when assayed at 10 and 20 °C, respectively (Fig. 4c). At the temperature of 0 °C, invertase activity of 19.7% was even retained (Fig. 4c). Half-lives of the enzyme were approximately 30 and 10 min when assayed at 30 and 37 °C, respcetively (Fig. 4d).

Addition of 1.0 mM MnSO_4_ and β-mercaptoethanol slightly enhanced the activity of rInvHJ14, but addition of CuSO_4_, HgCl_2_ and SDS strongly or completely inhibited the enzyme activity. The presence of other metal ions and chemical reagents had little or no effect on the enzyme activity (Table S1).

**Figure 5:**
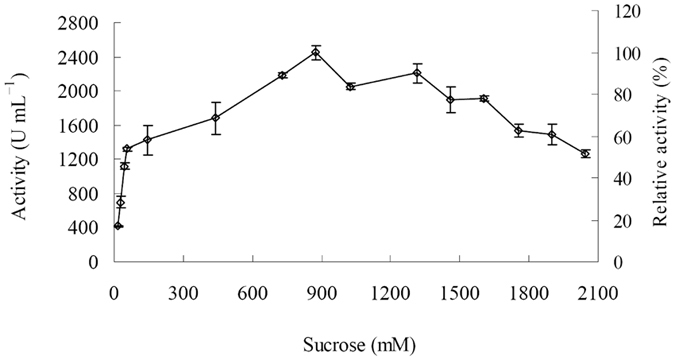
The effect of sucrose on purified rInvHJ14. *Error bars* represent the means ± SD (n = 3).

The activity of rInvHJ14 increased with the increase in sucrose concentration from 14.6 mM to 876.4 mM (Fig. 5). Then, rInvHJ14 was inhibited by the substrate at concentration of higher than 876.4 mM (Fig. 5). rInvHJ14 preserved approximately 50% of its highest activity in the presence of 2045.0 mM sucrose (Fig. 5).

### Kinetic and thermodynamic characterization

**Figure 6:**
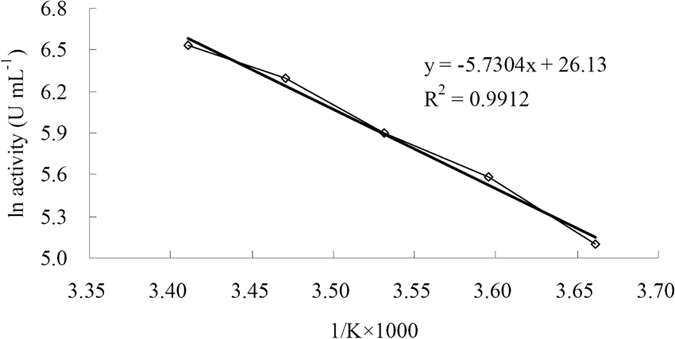
Arrhenius plot for the determination of *E*_a_ for sucrose hydrolysis by purified rInvHJ14.

From the Arrhenius plots (Fig. 6), the *E*_a_ value for hydrolysis of sucrose by rInvHJ14 was 47.6 kJ mol^−1^. The *Q*_10_ value (20 °C and 30 °C) for the hydrolysis of sucrose by rInvHJ14 was 1.9. Other kinetic and thermodynamic values are summarized in Table 1.

### Transglycosylation activity

Self-condensation or transglycosylation product was not observed for rInvHJ14 in this study (Fig. S3).

## Discussion

rInvHJ14 shows typical properties of low-temperature-active enzymes as its optimal activity is 30–32.5 °C, it has high catalytic activity at 0 and 10 °C, and it is thermolabile at 30 °C or higher temperatures^15^. Compared with rInvHJ14, most invertases show properties of mesophilic or thermophilic enzymes as their optimal activities are at temperatures of equal to or higher than 45 °C, they are thermostable at 50 °C or higher than 50 °C, and they show no activity or less than 10% relative activity at 10 °C^2^,^3^,^7^,^8^,^12^,^18^,^24^,^28^,^29^,^30^,^31^,^32^,^33^,^34^,^35^. The kinetic and thermodynamic properties of rInvHJ14 further indicate that the invertase is a low-temperature-active enzyme. Many low-temperature-active enzymes have lower *E*_a_ values than their more thermostable homologs because *E*_a_ decreases with a decrease in ΔG^*^ or a decrease in energy barriers which allow easy conformational changes of enzymes during catalysis at low temperatures^36^,^37^. The *E*_a_ and ΔG^*^ values for hydrolysis of sucrose by rInvHJ14 was 47.6 kJ mol^−1^ and 57.6 kJ mol^−1^, respectively, which are lower than the values for some thermostable invertases, such as *Saccharum officinarum* L. invertase showing an optimal temperature of 55 °C with an *E*_a_ value of 55.3 kJ mol^−1^ and a ΔG^*^ value of 71.2 kJ mol^−1^ 18, *Fusarium* sp. invertase showing an optimal temperature of 50 °C and an *E*_a_ value of 72.4 kJ mol^−1^ 35, and *Oryza sativa* invertase showing an *E*_a_ value of 100.8 kJ mol^−1^ 9. The majority of low-temperature-active enzymes have higher *K*_m_ and *k*_cat_ values than their thermostable counterparts in order to decrease the activation free-energy barrier^36^. rInvHJ14 has higher *K*_m_ (62.9 mM) and *k*_cat_ (746.2 s^−1^) values than some thermostable invertases, such as *Elsholtzia haichowensis* invertase showing an optimal temperature of 70 °C with a *K*_m_ value of 2.68 mM and a *k*_cat_ value of 469.17 s^−1^ 38, *Kluyveromyces marxianus* invertase showing an optimal temperature of 50–55 °C with a *K*_m_ value of 2.5 mM and a *k*_cat_ value of 301 s^−1^ 12, and many microbial invertases with *K*_m_ values of lower than 60 mM as reviewed previously^2^.

Catalytic domains of many low temperature-active enzymes present more number of glycine residues but less number of proline residues especially in loops than their more thermostable homologs because glycine residues can provide localized chain mobility but proline residues restrict backbone rotations^15^,^16^. That will generate enhanced flexible region surrounding the catalytic site and finally allow catalysis of enzymes at low temperatures^15^,^16^. As shown in Fig. 1, InvHJ14 has more number or higher frequency of glycine residues but less number or lower frequency of proline residues in the catalytic domain from conserved HXXXXXXXXNDPNG to ECXXX than some mesophilic and thermophilic invertases. The number or frequency of glycine residues in the catalytic domain of InvHJ14 is approximately 2-fold compared with that of the invertase InvSC. On the contrary, the numbers or frequencies of proline residues in the catalytic domains of the invertases InvIB, InvUB, InvSL from *Solanum lycopersicum* (AAA34132)^39^ and InvEH from *E. haichowensis* (AFV59227)^38^ are around 2-fold compared with that of InvHJ14. And the numbers or frequencies of proline residues in the catalytic domains of the invertases InvSC and InvTM are approximately 1.5-fold compared with that of InvHJ14. Notably, proline residues in most mesophilic and thermophilic invertases increase significantly in the segment corresponding to V149–G155 of InvHJ14 as shown in Fig. 1. These amino acid residues in the segment build a loop near the predicted transition-state stabilizer D162 (Fig. 2). Consequently, the increased number of glycine residues combined with the decreased number of proline residues allow easier local conformational changes of InvHJ14 and finally contribute to low-temperature adaptation of the enzyme.

The amplitude of the movement between secondary structures can be increased by longer loop then result in the higher structure flexibility^16^. InvHJ14 has an increased number of amino acid residues from N210 to F215 compared with some mesophilic and thermophilic invertases (Fig. 1). These amino acid residues build a longer loop which is close to the catalytic E220 (Fig. 2). As such, the proper plasticity close to the catalytic site enables rInvHJ14 maintaining high catalytic activity at low temperatures accompanied by thermolability at intermediate and high temperatures.

rInvHJ14 is an alkaline invertase as it shows activity and stability at alkaline conditions. To our knowledge, alkaline invertases have been reported only from plants and cyanobacteria^2^,^4^,^5^,^20^,^21^,^22^,^23^,^24^. Most invertases produced by microorganisms worked optimally in the range of pH 2.9–6.2^2^. As such, the study is the first to report an alkaline invertase isolated from eubacteria. Compared with their acidic counterparts, alkaline enzymes have a larger positively charged surface to keep them active and stable under alkaline condition, such as the alkaline β-mannanase from *Humicola insolens* Y1^40^ and α-galactosidase from *Streptomyces* sp. HJG4^37^. In this study, structure analysis found that the catalytic pockets of acidic InvSC, InvIB, InvTM, InvBL and InvUB were almost negatively charged while that of alkaline InvHJ14 was mostly positively charged. Therefore, a positively charged catalytic pocket is presumed to be a factor for alkaline adaptation of the GH 32 invertase rInvHJ14.

Furthermore, enzymatic hydrolysis of sucrose is preferable in the production of high-quality inverted syrups compared with acidic hydrolysis^41^. A high sucrose content; even as high as 2 M, is used in the industrial application^41^. As such, the identification of an invertase that is active in the presence of 2 M sucrose is relevant. It has been reported that the commercial invertase from *S. cerevisiae* displayed approximately 30% of its highest activity at 2 M sucrose^42^. The invertases from a metagenomic library and *Aspergillus niger* show approximately 50%^24^ and 30%^10^ of their highest activities at 1 M sucrose, respectively. In the presence of 2 M sucrose, the yeast *Candida guilliermondii* MpIIIa invertases INV3a-N and INV3a-D present nearly 50% and 10% of their highest activities, respectively^7^. Similar to INV3a-N, rInvHJ14 remained approximately 50% of its highest activity in the presence of 2045.0 mM sucrose. The big differences between INV3a-N and rInvHJ14 are that INV3a-N is an acidic and thermophilic invertase^7^. Together, rInvHJ14 may be a potential candidate for enzymatic hydrolysis of sucrose in the production of high-quality inverted syrups.

In conclusion, a GH 32 invertase was isolated from an eubacteria—*Bacillus* sp. HJ14. Biochemical characterization revealed that the enzyme was a novel low-temperature-active, alkaline and sucrose-tolerant invertase. These characteristics make the invertase suitable for versatile applications, especially in the food industry. Moreover, molecular characterization of the enzyme contributes to a better understanding of the structure–function relationships of low-temperature-active and alkaline invertases.

## Materials and Methods

### Strains, vectors and reagents

The details of isolation, identification and preservation of *Bacillus* sp. HJ14 were described in our previous study^43^.

*E. coli* BL21 (DE3) and *pEASY*-E2 vector (TransGen, Beijing, China) were used for gene expression. Ni^2+^-NTA agarose (Qiagen, Valencia, CA, USA) was used to purify the recombinant protein. Genomic DNA and plasmid isolation kits were purchased from Tiangen (Beijing, China). DNA polymerases (*Taq* and *Pyrobest*) and dNTPs were purchased from TaKaRa (Otsu, Japan). Sucrose, birchwood xylan, beechwood xylan, *p*-nitrophenyl-β-d-xylopyranoside, inulin from dahlia tubers (I3754), raffinose, starch, d-glucose, d-galactose, d-fructose and d-xylose were purchased from Sigma−Aldrich (St. Louis, MO, USA). Wheat flour arabinoxylan (P-WAXYM) was purchased from Megazyme (Wicklow, Ireland). Stachyose and sucralose were purchased from Tokyo Chemical Industry (Tokyo, Japan). Fructooligosaccharides set including kestose, nystose and fructofuranosylnystose was purchased from Wako Pure Chemical (Osaka, Japan). Levan from *Zymomonas mobilis* was purchased from Advanced Technology & Industrial (Hong Kong, China), Isopropyl-β-d-1-thiogalactopyranoside (IPTG) was purchased from Amresco (Solon, OH, USA). Silica gel G plates were purchased from Haiyang (Qingdao, China). All chemicals were of analytical grade.

### Gene cloning and sequence analysis

The genome of HJ14 was sequenced on a Hiseq 2000 sequencer (Illumina) and genomic data was analyzed on a NF supercomputing server (Inspur, Shandong, China) in our lab. Other details are as described in our previous study^43^.

Genes from the draft genome of HJ14 were predicted by the tools GeneMark.hmm 2.8 (http://exon.gatech.edu/GeneMark/gmhmm2_prok.cgi) combined with local BLAST 2.2.25^44^. The identity values of DNA and protein sequences were obtained from the BLASTN and BLASTP (http://www.ncbi.nlm.nih.gov/BLAST/) results, respectively. The signal peptide and catalytic domain were predicted by SignalP (http://www.cbs.dtu.dk/services/SignalP/) and InterPro online tool (http://www.ebi.ac.uk/interpro/), respectively.

### Structure analysis

Tertiary structures of invertases were predicted by homology modeling using SwissModel (http://swissmodel.expasy.org/). Spatial distributions of electrostatic potential of tertiary structures were calculated using the Discovery Studio 2.5 software (Accelrys, San Diego, CA, USA).

### Heterologous expression

The InvHJ14-encoding gene (*invHJ14*) was amplified by PCR using *Pyrobest* DNA polymerase and the primer set r*invHJ14*EF (ATGACAACTACTGACGCAGC) and r*invHJ14*ER (TTGATCTGCTTCTCTTTGTAAAT), and then the resulting PCR product was added base A at the 5′ terminal using *rTaq* DNA polymerase. The recombinant plasmid, designated *pEASY-invHJ14*, was constructed with *invHJ14* and *pEASY*-E2 vector through T-A ligation and transformed into *E. coli* BL21 (DE3) competent cells. The positive transformant harboring *pEASY-invHJ14* was identified by PCR analysis and confirmed by DNA sequencing. The overnight culture of positive transformant was inoculated (1:100 dilutions) into fresh Luria–Bertani medium containing 100 μg mL^−1^ ampicillin, and incubated at 37 °C. Upon reaching an OD_600nm_ of 0.7, 0.7 mM IPTG was added to the broth to induce enzyme expression at 20 °C for further 20 h.

### Purification and identification of recombinant enzyme

*E. coli* cells were collected by centrifugation at 10,000 × *g* at room temperature for 5 min. The resulting cell pellet was resuspended in sterilized, ice-cold buffer A (20 mM Tris–HCl, 0.5 M NaCl, pH 7.2), and sonicated on ice with 100 short bursts for 4 s each at a power output of 150 W. The suspension was centrifuged at 10,000 × *g* at room temperature for 5 min then the precipitation was removed. The supernatant was collected and passed over Ni^2+^-NTA agarose gel columns previously equilibrated with buffer A. Stepwise elution was carried out with buffer A containing 0, 20, 40, 60, 80, 100, 200, 300 and 500 mM imidazole, in that order. Eluted fractions containing enzyme activity were collected and stored at −20 °C until used for subsequent analyses.

For determination of protein purity after Ni^2+^-NTA purification, eluted fractions were loaded for sodium dodecyl sulfate–polyacrylamide gel electrophoresis (SDS–PAGE) using 12% running gels. The purified protein in the SDS–PAGE gel was identified using matrix-assisted laser desorption/ionization time-of-flight mass spectrometry (MALDI–TOF MS) performed by Tianjin Biochip (Tianjin, China). The protein concentration was determined with Bradford method using bovine serum albumin as the standard^45^.

### Enzyme assay and substrate specificity

The sucrose hydrolysis reactions involved incubations at 37 °C for 10 min with a reaction mixture containing 100 μL of appropriately diluted rInvHJ14 and 900 μL of McIlvaine buffer (pH 8.0) containing 0.5% (w/v) sucrose. The amount of reducing sugars released was determined by the 3,5-dinitrosalicylic acid (DNS) reagent at 540 nm^46^. One activity unit (U) was defined as the amount of enzyme that produces 1.0 μmol equimolar mixture of glucose and fructose per minute under the above assay conditions unless otherwise noted.

Substrate specificity assay was performed at 37 °C in McIlvaine buffer (pH 8.0) using 0.5% (w/v) sucralose, raffinose, stachyose, levan, inulin, starch, birchwood xylan, beechwood xylan, wheat flour arabinoxylan or fructooligosaccharides mixture as substrate instead of sucrose. Enzyme catalysis on *p*-nitrophenyl-β-d-xylopyranoside was assayed using the method described in the previous study^43^.

### Biochemical characterization

Biochemical characterization of purified rInvHJ14 was determined using sucrose as substrate unless otherwise noted.

The effects of pH and temperature on rInvHJ14 were determined at pH 7.0–10.0 and 0–50 °C, respectively. pH and temperature stabilities of rInvHJ14 were studied respectively by keeping the enzyme solution at 37 °C for 60 min at different pH values (pH 5.0–11.0) and by keeping the enzyme solution at pH 8.0 for 1–60 min at different temperatures (30–50 °C). Residual activities of these pre-incubated samples were then determined under standard assay conditions. The buffers used were McIlvaine buffer for pH 5.0–8.5 and 0.1 M glycine–NaOH for pH 9.0–11.0.

Various metal ions and chemical reagents were individually added to the reaction solution to investigate their effects on rInvHJ14. These metal ions and chemical reagents were 1.0 mM (final concentration) NaCl, KCl, CaCl_2_, CoCl_2_, NiSO_4_, CuSO_4_, MgSO_4_, MnSO_4_, ZnSO_4_, Pb(CH_3_COO)_2_, HgCl_2_, FeCl_3_, EDTA, β-mercaptoethanol and SDS, and 1.0% (v/v) Tween 80 and Triton X-100. Any precipitation was removed by centrifugation before measuring the absorption.

In order to determine whether the activity of rInvHJ14 was inhibited by sucrose at high final concentrations, the enzyme was incubated in the reaction mixture with 14.6–2045.0 mM sucrose. Enzyme activity was then determined under standard assay conditions.

### Kinetic and thermodynamic characterization

*K*_m_, *V*_max_ and *k*_cat_ values of purified rInvHJ14 were determined using 1.5–36.5 mM sucrose as substrate in McIlvaine buffer (pH 8.0) at 10, 20 and 30 °C. The data were plotted according to the Michaelis–Menten method using the computer software GraphPad Prism (GraphPad Software, San Diego, CA, USA).

The activation energy (*E*_a_), free energy of activation (ΔG^*^), enthalpy of activation (ΔH^*^), entropy of activation (ΔS^*^) and temperature coefficient (*Q*_10_) for rInvHJ14 were calculated using the equations as described in previous study^47^.

### Transfructosylating reactions

In order to determine whether rInvHJ14 possessed self-condensation activity, the enzyme (approximately 0.5 U mL^−1^) was incubated in the reaction mixture with 146 or 584 mM sucrose at 20 °C and pH 8.0 for 17 h. Transglycosylation reactions were carried out at 20 °C and pH 8.0 for 17 h using 146 mM sucrose as the donor with d-galactose and d-xylose as the acceptors at 555 mM and 666 mM, respectively. Transglycosylation products were analyzed by thin layer chromatography (TLC) as previously described^37^.

### Accession number

Nucleotide sequence of *invHJ14* was deposited in GenBank under the accession number KT943473.

## Additional Information

**How to cite this article**: Zhou, J. *et al.* Characterization of a novel low-temperature-active, alkaline and sucrose-tolerant invertase. *Sci. Rep.* **6**, 32081; doi: 10.1038/srep32081 (2016).

## Supplementary Material

Supplementary Information

## Figures and Tables

**Table 1:** Kinetic and thermodynamic characterization of purified rInvHJ14 toward sucrose.

Parameters	10 °C	20 °C	30 °C
*K*_m_ (mM)	50.3	57.6	62.9
*V*_max_ (μmol min^−1^ mg^−1^)	231.7	599.7	771.5
*k*_cat_ (s^−1^)	224.1	580.0	746.2
*k*_cat_*/K*_m_ (mM^−1^ s^−1^)	4.4	10.1	11.9
ΔG^*^ (kJ mol^−1^)	56.5	56.2	57.6
ΔH^*^ (kJ mol^−1^)	45.3	45.2	45.1
ΔS^*^ (J mol^−1^ K^−1^)	−39.4	−37.6	−41.1
