# Supplementary material for: Characterization of a novel low-temperature-active, alkaline and sucrose-tolerant invertase
Source: Sci Rep. 2016 Aug 24;6:32081. doi: 10.1038/srep32081 (PMC4995436; doi:10.1038/srep32081)
Supplement: Supplementary Information [file srep32081-s1.pdf]

Characterization of a novel low-temperature-active, alkaline and sucrose-tolerant invertase

Junpei Zhou[1,2,3,4,*], Limei He[2,*], Yajie Gao[2], Nanyu Han[1,2,3,4], Rui Zhang[1,2,3,4], Qian Wu[1,2,3,4], Junjun Li[1,2,3,4], Xianghua Tang[1,2,3,4], Bo Xu[1,2,3,4], Junmei Ding[1,2,3,4] & Zunxi Huang[1,2,3,4]

[1]Engineering Research Center of Sustainable Development and Utilization of Biomass Energy, Ministry of Education, Yunnan Normal University, Kunming, 650500, People's Republic of China

[2]College of Life Sciences, Yunnan Normal University, Kunming, 650500, People's Republic of China

[3]Key Laboratory of Yunnan for Biomass Energy and Biotechnology of Environment, Yunnan, Kunming, 650500, People's Republic of China

[4]Key Laboratory of Enzyme Engineering, Yunnan Normal University, Kunming, 650500, People's Republic of China

[*]J.Z. and L.H. contributed equally to this work

Correspondence and requests for materials should be addressed to Z.H. (Tel.: +86 871 65920830; Fax: +86 871 65920952; e-mail: huangzunxi@163.com)

Table S1. Effects of 1.0 mM metal ions and chemical reagents on the activity of purified rInvHJ14.

Substance	Relative activity (%)[a]
None	100.0 ± 0.4
$MnSO_4$	123.2 ± 6.2
$CaCl_2$	96.7 ± 4.7
$Pb(CH_3COO)_2$	95.9 ± 1.2
$CoCl_2$	93.9 ± 2.6
NaCl	90.1 ± 6.5
KCl	90.0 ± 2.5
$MgSO_4$	88.4 ± 5.4
$NiSO_4$	85.9 ± 5.1
$ZnSO_4$	80.8 ± 3.3
$FeCl_3$	74.0 ± 2.1
$CuSO_4$	13.6 ± 0.6
$HgCl_2$	0.0
β-Mercaptoethanol	129.1 ± 3.6
Tween 80 [0.5% (v/v)]	97.4 ± 2.9
EDTA	96.8 ± 0.7
Triton X-100 [0.5% (v/v)]	95.1 ± 1.5

SDS	0.0

[a] Values represent the means ± SD (n = 3) relative to the untreated control sample.

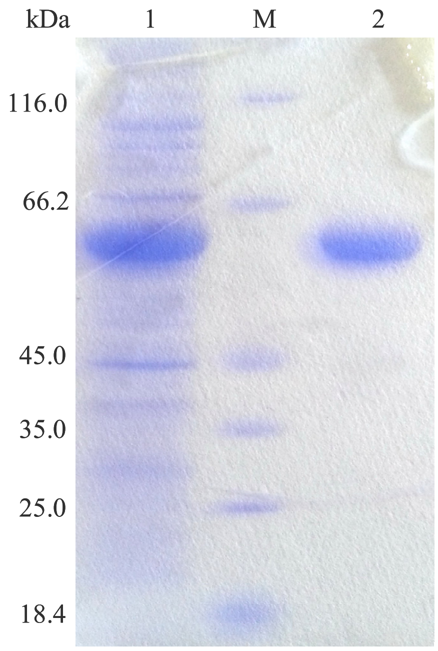

Figure S1. SDS–PAGE analysis of rInvHJ14.

Lanes: *M*, protein molecular weight marker; *1*, cell extract of an induced positive transformant harboring *pEASY-invHJ14*; *2*, purified rInvHJ14.

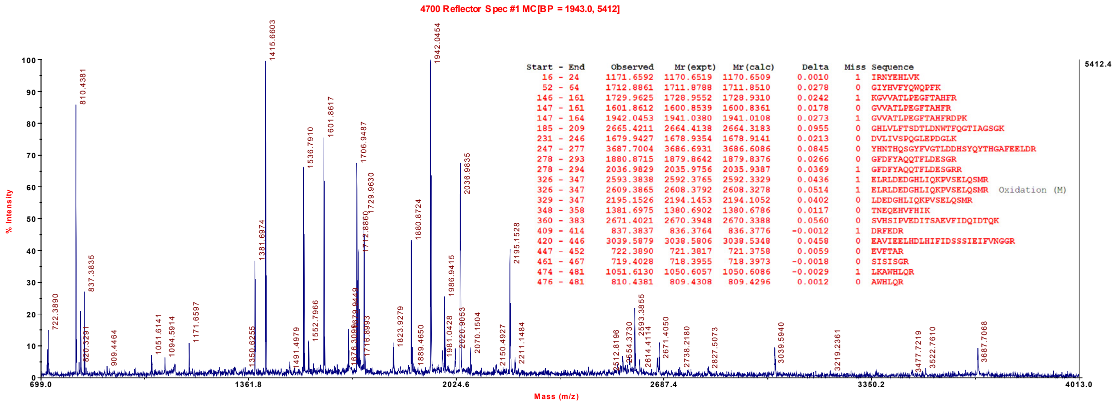

Start - End	Observed	Mr(expt)	Mr(calc)	Delta	Miss	Sequence
16 - 24	1171.6592	1170.6519	1170.6509	0.0010	1	IRNYEHLVK
52 - 64	1712.8861	1711.8788	1711.8510	0.0278	0	GIYHVFYQWQPFK
146 - 161	1729.9625	1728.9552	1728.9310	0.0242	1	KGVVATLPEGFTAHFR
147 - 161	1601.8612	1600.8539	1600.8361	0.0178	0	GVVATLPEGFTAHFR
147 - 164	1942.0453	1941.0380	1941.0108	0.0273	1	GVVATLPEGFTAHFRDPK
185 - 209	2665.4211	2664.4138	2664.3183	0.0955	0	GHLVLFTSDTLDNWTFQGTIAGSGK
231 - 246	1679.9427	1678.9354	1678.9141	0.0213	0	DVLIVSPQGLEPDGLK
247 - 277	3687.7004	3686.6931	3686.6086	0.0845	0	YHNTHQSGYFVGTLDDHSYQYTHGAFEELDR
278 - 293	1880.8715	1879.8642	1879.8376	0.0266	0	GFDFYAQQTFLDESGR
278 - 294	2036.9829	2035.9756	2035.9387	0.0369	1	GFDFYAQQTFLDESGRR
326 - 347	2593.3838	2592.3765	2592.3329	0.0436	1	ELRLDEDGHLIQKPVSELQSMR
326 - 347	2609.3865	2608.3792	2608.3278	0.0514	1	ELRLDEDGHLIQKPVSELQSMR Oxidation (M)
329 - 347	2195.1526	2194.1453	2194.1052	0.0402	0	LDEDGHLIQKPVSELQSMR
348 - 358	1381.6975	1380.6902	1380.6786	0.0117	0	TNEQEHVFHIK
360 - 383	2671.4021	2670.3948	2670.3388	0.0560	0	SVHSIPVEDITSAEVFIDQIDTQK
409 - 414	837.3837	836.3764	836.3776	-0.0012	1	DRFEDR
420 - 446	3039.5879	3038.5806	3038.5348	0.0458	0	EAVIEELHDLHIFIDSSSIEIFVNGGR
447 - 452	722.3890	721.3817	721.3758	0.0059	0	EVFTAR
461 - 467	719.4028	718.3955	718.3973	-0.0018	0	SISISGR
474 - 481	1051.6130	1050.6057	1050.6086	-0.0029	1	LKAWHLQR
476 - 481	810.4381	809.4308	809.4296	0.0012	0	AWHLQR

Figure S2. The MALDI–TOF MS spectrum of the single band of approximately 58 kDa cut from the SDS–PAGE gel of purified rInvHJ14.

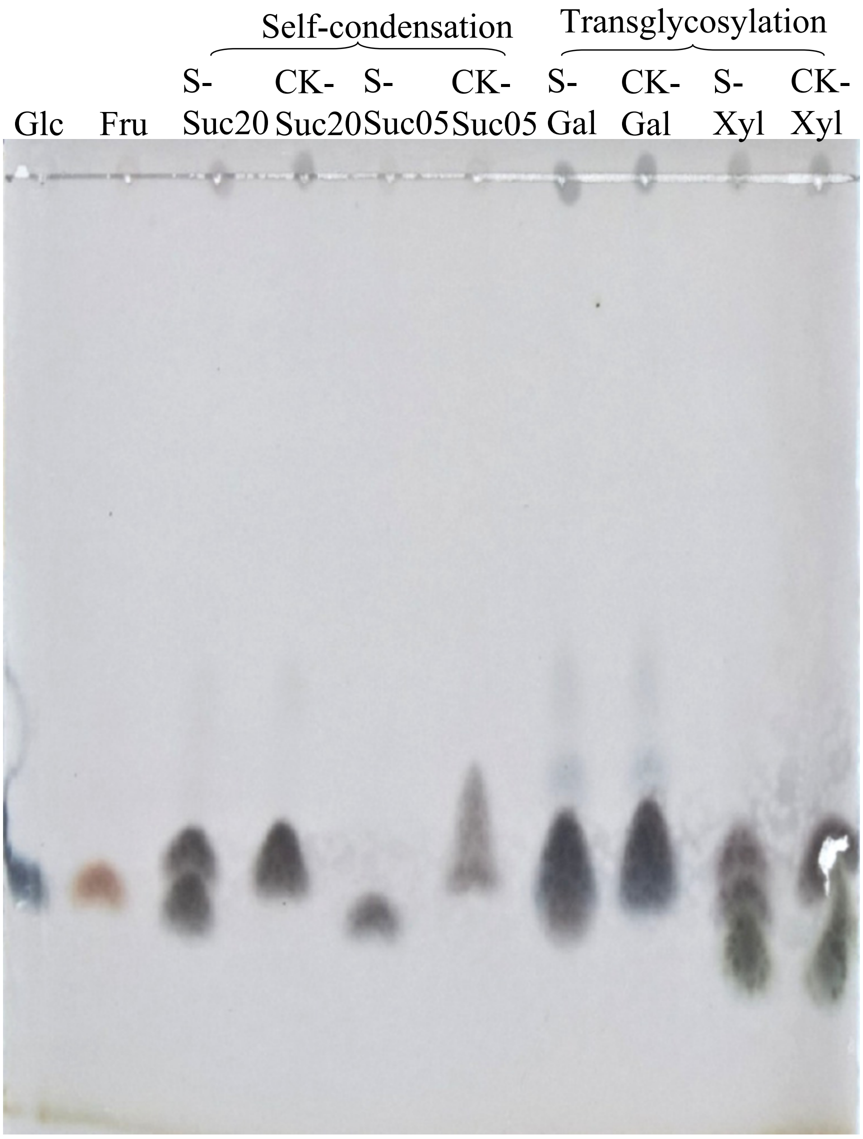

Figure S3. Thin layer chromatography analysis of transglycosylation products.

Lanes: *Glc* and *Fru* represent glucose and fructose, respectively; *CK-X* and *S-X* represent control and experimental groups, respectively, and *X* represents galactose (*Gal*), xylose (*Xyl*) or sucrose at the concentrations of 50 mg ml^{-1} (146 mM; *Suc05*) or 200 mg ml^{-1} (584 mM; *Suc20*).

>*invHJ14* (KT943473)

ATGACAACTACTGACGCAGCACTTCGTCAAAAGATAGCAGACCGTATTCGAAA

CTATGAGCATTTGGTCAAAAAGGATGTGTACCGGCAGCATTTTCATTTGATGCC

GCCAGTCGGTCTTTTAAATGATCCGAATGGGCTGATTCAGTGGAAAGGAATTT

ACCACGTCTTTTATCAGTGGCAGCCATTCAAAACGGGGCATGGTGCAAAATTC

TGGGGGCATTACACATCGACAGATCTAGTCAATTGGCAGCACGAAGAAGCCGC

TCTTGCCCCAAGTGATTGGTTTGATCAAAACGGCTGTTATTCTGGCAGTGCATT

TATAGACGATGGACAGATGCATGTGATGTACACTGGAAACGTTCGGGATGAAC

AAGGGAACCGTGAGACGTATCAATGTTTAGCTGTTTCGGAAGATGGTATTCAC

TTTCAGAAAAAAGGTGTCGTGGCAACACTGCCGGAAGGGTTCACTGCTCATTT

TCGAGATCCAAAGGTATGGAAGCGAAATGGCCAGTGGTACATGGTCCTTGGTG

CACAAAGCTTAGACCTGAAGGGACACCTTGTCTTATTTACCTCTGATACGTTGG

ATAATTGGACGTTTCAAGGCACGATTGCAGGCAGCGGAAAGAATGGTTTAGAC

AATTTCGGCTATATGTGGGAATGTCCAGACCTGTTTGAATTAGACGGCCGGGAT

GTATTGATTGTGTCACCGCAAGGGCTAGAGCCGGATGGCTTAAAATATCACAA

CACACATCAATCAGGCTATTTTGTTGGCACATTGGATGATCATTCCTATCAATAT

ACACACGGAGCGTTTGAGGAGCTGGACCGCGGCTTTGACTTCTATGCGCAGC

AAACCTTCCTAGACGAATCAGGCAGACGACTTTTAATCGGGTGGATGGGGGTG

CCTGATCAAGGAGAAGAACATCATCCGACCATTTCTTATCAATGGATACACTGC

CTCACGATCCCGAGAGAGCTCCGTTTAGATGAGGACGGACATCTGATTCAAAA

ACCAGTAAGTGAACTTCAATCCATGCGAACCAATGAACAAGAGCATGTTTTTC

ATATTAAACGTTCTGTTCATTCCATCCCTGTAGAGGATATTACGAGCGCAGAGG

TATTCATTGATCAAATTGACACACAAAAAGGGTTTGAATGCTGTATCCGTGCAG

CAGCACGCATCATCTATGATAAAGAGGAAGGGAAGCTGACATTAGAGCGGGAT

CGATTTGAAGATCGATCAAAGGAAGTTCGTGAAGCAGTCATAGAGGAATTACA

CGACCTTCATATCTTTATCGACTCATCGTCAATTGAGATTTTTGTAAACGGAGGC

CGAGAAGTTTTTACTGCACGTTACTTTCCTTCCCCGGGAAATAAATCGATCTCC

ATCAGTGGCAGAAATGAAACAAAGCTGAAACTGAAGGCATGGCATTTACAAA

GAGAAGCAGATCAATGA

>InvHJ14 translated from *invHJ14*

MTTTDAALRQKIADRIRNYEHLVKKDVYRQHFHLMPPVGLLNDPNGLIQWKGIY

HVFYQWQPFKTGHGAKFWGHYTSTDLVNWQHEEAALAPSDWFDQNGCYSGSA

FIDDGQMHVMYTGNVRDEQGNRETYQCLAVSEDGIHFQKKGVVATLPEGFTAHF

RDPKVWKRNGQWYMVLGAQSLDLKGHLVLFTSDTLDNWTFQGTIAGSGKNGL

DNFGYMWECPDLFELDGRDVLIVSPQGLEPDGLKYHNTHQSGYFVGTLDDHSY

QYTHGAFEELDRGFDFYAQQTFLDESGRRLLIGWMGVPDQGEEHHPTISYQWIH

CLTIPRELRLDEDGHLIQKPVSELQSMRTNEQEHVFHIKRSVHSIPVEDITSAEVFID

QIDTQKGFECCIRAAARIIYDKEEGKLTLERDRFEDRSKEVREAVIEELHDLHIFIDS

SSIEIFVNGGREVFTARYFPSPGNKSISISGRNETKLKLKAWHLQREADQ*
